# Life events and depression among children and adolescents in southwest China: a two-stage moderated mediation model of social support and cognitive styles

**DOI:** 10.1186/s12888-022-04454-5

**Published:** 2022-12-21

**Authors:** Bicheng Gao, Kuiliang Li, Ju Liu, Xuan Liu, Jingxuan Zhang, Chen Xu, Yuhao He, Zhengzhi Feng, Mengxue Zhao

**Affiliations:** 1 Department of Military Psychology, Faculty of Medical Psychology, Army Medical University (Third Military Medical University), Chongqing, People’s Republic of China; 2 Department of Psychology and Behavioral Sciences, Zhejiang University, Hangzhou, Zhejiang People’s Republic of China; 3 Department of Psychology, School of Philosophy, WuHan University, Wuhan, Hubei People’s Republic of China; 4 Faculty of Medical Psychology, Army Medical University (Third Military Medical University), Chongqing, People’s Republic of China; 5 Dapartment of Foreign Languages, College of Basic Medical Sciences, Army Medical University (Third Military Medical University), Chongqing, People’s Republic of China; 6 Army Health Service Training Base, Army Medical University (Third Military Medical University), Chongqing, People’s Republic of China

**Keywords:** Depression, Life events, Social support, Cognitive styles, Children and adolescents

## Abstract

**Background:**

According to data from the National Health Commission in 2018, more than 30 million children and adolescents in China suffered from mental health problems of varying degrees, with depression accounting for the largest proportion. Life events occur at every stage of child and adolescent development. Many studies have found a relationship between life events and depression in children and adolescents, but few studies have further explored the mediating and moderating variables that influence this relationship. Based on theoretical and empirical research on social support and cognitive styles, this study established a two-stage moderated mediating model to test whether social support mediates life events to depression, and cognitive style moderates the two mediating pathways.

**Methods:**

We recruited 3540 participants from primary and secondary schools in Chongqing and collected 2814 valid data. All the participants completed self-report measures of life events, depression, social support and cognitive styles. The moderated mediation model was examined using SPSS PROCESS model 58.

**Results:**

(1) There were significant positive correlations between life events and depression. (2) Social support mediates the relationship between life events and depression in children and adolescents. (3) Cognitive style moderates life events to social support and social support to depression.

**Limitations:**

This is a cross-sectional study and the questionnaire is self-reported.

**Conclusions:**

In children and adolescents, life events can influence depression through the mediating role of social support and cognitive styles could moderate its two mediating pathways.

## Introduction

According to the latest demographic statistics, the number of children and teenagers aged 7–19 in China accounts for more than 10% of the total population and their health will largely determine the future of the country. A report from the National Health Commission in 2018 indicated that more than 30 million children and adolescents suffered from mental health problems to different degrees [1], with depression accounting for the largest proportion. Depression in children and adolescents is one of the most common mental health problems in recent years. Statistically, depression is the third leading cause of disability among adolescents worldwide. A meta-analysis of Chinese adolescents and children showed that 19.8% to 24.3% of them had depressive symptoms [2]. Depression in children and adolescents not only interferes with children's normal development and prevents children from reaching their potential [3], but also causes memory impairment [4] and increases risky suicidal behaviors [5], substance abuse [6] and other emotional disorders in adulthood [7]. Studies have found that adolescents and children are susceptible to major depression [8]. The effects of depression not only last for the whole adolescence [9], but also generate an impact on psychological development in adulthood [10].

Many factors may account for the mental health issues of children and adolescents. Previous studies have shown that depression in children and adolescents was associated with financial status [11]. This is a strong influencing factor in the case of left-behind children and adolescents [12]. In the process of industrialization and urbanization, a large number of young and middle-aged rural laborers packed their luggage to land a job in cities for better financial circumstances. However, because of the dualistic social structure in urban and rural areas, especially the institutional restrictions on education and social security, migrant workers might be forced to leave their children behind in rural areas. Left-behind children, aged between 7 to 17, usually live with one of their parents or other caregivers and one or both of their parents worked in urban areas in the past six months or more [13]. The left-behind phenomenon was found to cause more behavioral and psychological problems for children [14, 15]. Studies have shown that left-behind children report more depressive symptoms than non-left-behind children [16]. Compared with non-left-behind children, left-behind children experienced more life stress and life events [17]. Due to the lack of parental companionship, left-behind children often had to take care of the elderly at home. Meanwhile, left-behind children also faced problems such as excessive housework, malnutrition, limited social interactions and low life satisfaction [18]. Moreover, as left-behind children lack family education, driven by their strong sense of inferiority, they are more inclined to avoid themselves in peer communication and show worse academic performance [19].

For the study of children’s mental health issues, different factors might be relevant to different countries or regions. The purpose of our study is to investigate the mental health problems of children and adolescents and their influencing factors in Chongqing municipality, the biggest municipality in China. Chongqing is located in mountainous southwest China and its economy is relatively underdeveloped. A large number of young workers pack their luggage to land a job in other more well-developed urban cities while causing a series of social problems arising from their left-behind children. A survey of children's mental health in Chongqing found that up to 14.5 percent of children and adolescents had symptoms of depression. It is in this context that the research on the phenomenon of depression among children and adolescents in Chongqing is warranted.

### Life events within the context of depression

Life events refer to events or situations that threaten, damage or challenge an individual's mental and physical abilities [20]. Children and adolescents, as they are growing up, will encounter many challenges including different physical and psychological changes at different ages [21]. Inexperience and failure to cope with these changes may turn out to be life events causing great stress. According to the psychological stress model, life events are important risk factors in the development of children and adolescents, and life events can affect individuals' physical and mental health as the stressors [22]. Researchers found that negative life events were a significant predictor of depression in children [23], and children who experienced more negative life events were more likely to have depressive episodes in the future [24]. In addition, stressful life events in childhood also increase the risk of developing mental disorders in adulthood [25]. It is still worthwhile to further explore the relationship between life events and depression as life events may lead to depression, and then depression itself may also lead to stressful events, thus forming a vicious circle [26, 27].

Most of the previous studies only conducted preliminary discussions on the relationship between life events and depression, but failed to carry an in-depth discussion on the possible mediating and moderating factors. As a less developed city in southwest China, Chongqing has a large number of left-behind children and adolescents [28], sufficient studies on the mental issues of children and adolescents in Chongqing are lacking, therefore, it is worthwhile to explore the mental health problems of children and adolescents in Chongqing. If the mediating and moderating factors affecting life events and depression can be identified among children and adolescents in Chongqing, the government and relevant institutions can take more targeted measures for better prevention and treatment of these mental problems.

### The potential mediating effect of social support

Perceived social support refers to an individual's evaluation of the quality and quantity of social connections [29]. Researchers have found that social environments and social relationships play a key role in mental illness through underlying physiological and psychological mechanisms [30]. There is a strong association between social support and depression, and evidence suggests that perceived social support has a protective effect on mental health [31]. In a study of social support for college students, researchers found that students with low levels of social support were 6 times more likely to have depressive symptoms than students with high levels of social support [32]. Another study of social support from friends also showed that social support reduces depressive symptoms [33]. There is also a strong correlation between social support and life events. In both children [34] and the elderly [35], life events were significantly correlated with social support. Meanwhile, a study found that social support plays a mediating role in the relationship between childhood maltreatment and depression [36]. According to Folkman and Lazarus' general model of stress [37], stress is caused by the interaction between the individual and the environment. If a person is not adapted to the living environment, the morbidity rate of stress may increase. Meanwhile, according to Tedeschi and Calhoun's cognitive theory, social support is a protective factor for post-traumatic growth and development [38]. Research has found that social support is an effective stress reducing tool, and according to stress buffering theory, perceived social support can reduce the negative effects of stimuli [39], avoid negative emotions [40], and protect their physical and mental health [41]. The study also found that social support mediated the relationship between perceived stress and mental health among black women. Thus, based on the above literature and theory, we assume that social support may be a mediator influencing the relationship between life events and depression in adolescents and children.

### The potential moderating effect of cognitive styles

Cognitive style is one of the known risk factors for depression. Beck's cognitive model of depression provides a detailed explanation for the influence of cognition on depression [42, 43]. The model suggests that individuals form latent cognitive schemata early in life, which are activated in response to environmental pressures associated with these schemata. In turn, the cognitive schema determines how information is processed and how events and stimuli are interpreted [44]. Individuals in this negative cognitive mode tend to participate in, encode and retrieve biased schema-consistent information and are more likely to ignore positive and neutral information [45]. According to the cognitive theory of depression, maladaptive thinking and negative life evaluation will further lead to the occurrence and development of depression [46]. Research has shown that certain cognitive styles (e.g., internal stable global attribution of negative events, cognitive distortions and despair) are central features of childhood depression [3]. Furthermore, scholars also found that the negative cognitive style would become highly stable in adolescents [47]. Based on the reformulated learned helplessness theory, we can see that cognitive styles will interact with stressful events in the adolescent population [48, 49]. Individuals with a depressive attribution style may become depressed after a negative event because they may make a depressive attribution to a negative event and there are two depression cognitive styles: a depressogenic inferential style about the self (self-orientation), and a depressogenic inferential style about consequences (consequence orientation) [48]. According to stress buffering theory, perceived social support reduces stress. Perceived social support, as a cognitive process, is the individual feeling towards outside support. Previous studies have found that cognitive reappraisal plays a moderating role in depressive symptoms and perceived social support in adults [50]. Therefore, we hypothesize that there may be a moderating effect of the cognitive style on negative life events and social support. That is, individuals with negative cognitive styles perceive less social support after life events. In a study on adolescents' perceived social ability and depression, the moderating effect of negative cognitive style was found [51]. Thus, the moderating effect of cognitive style may also appear in the second mediation pathway, that is, individuals with more negative cognitive styles perceive less social support, which leads to depression. As mentioned above, in our study, we believe that the cognitive style, as a moderator, can not only moderate life events and social support, but also moderate the relationship between social support and depression.

### The current study

Although some researchers have explored the relationship between life events and depression, few have analyzed the specific pathways between life events and depression in children and adolescents. Social support and cognitive style, as two variables closely related to depression, have been studied in the past, but there has been no study exploring the complex relationship between these variables and their possible mediating and moderating roles. At the same time, to our knowledge, we are the first study to use cognitive style as an influencing variable to moderate the two-stage mediation pathway, and we are the first study to fully consider the two dimensions of cognitive styles: self-orientation and consequence orientation. Individuals with self-orientation cognitive style tend to view themselves as flawed and deficient after the occurrence of negative events while people with consequence orientation are prone to view negative events as having many disastrous consequences [48].

Considering the severity of left-behind children and adolescents in Chongqing, it’s not realistic to change their experiences of negative life events, but increasing social support and improving their cognitive styles are feasible. This study will further explore the relationship between these four variables and provide a theoretical framework for the prevention and intervention of depressive symptoms in children and adolescents.

**Fig. 1:**
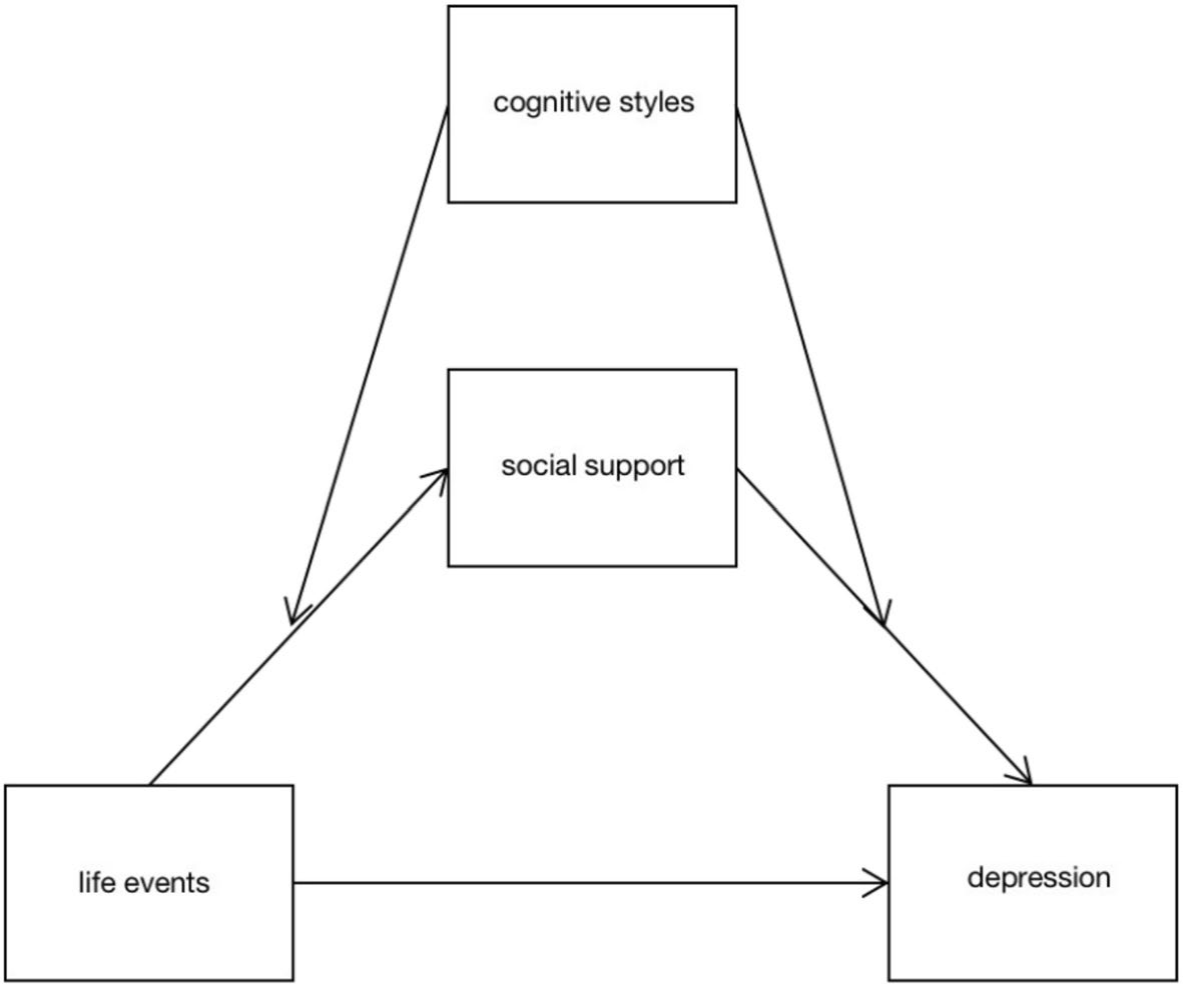
Conceptual framework

**Fig. 2:**
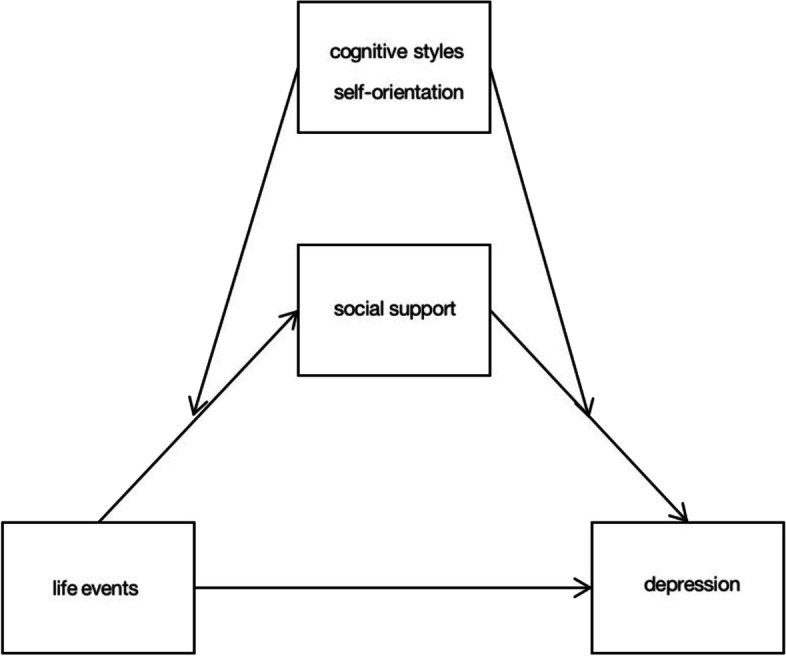
Conceptual framework

**Fig. 3:**
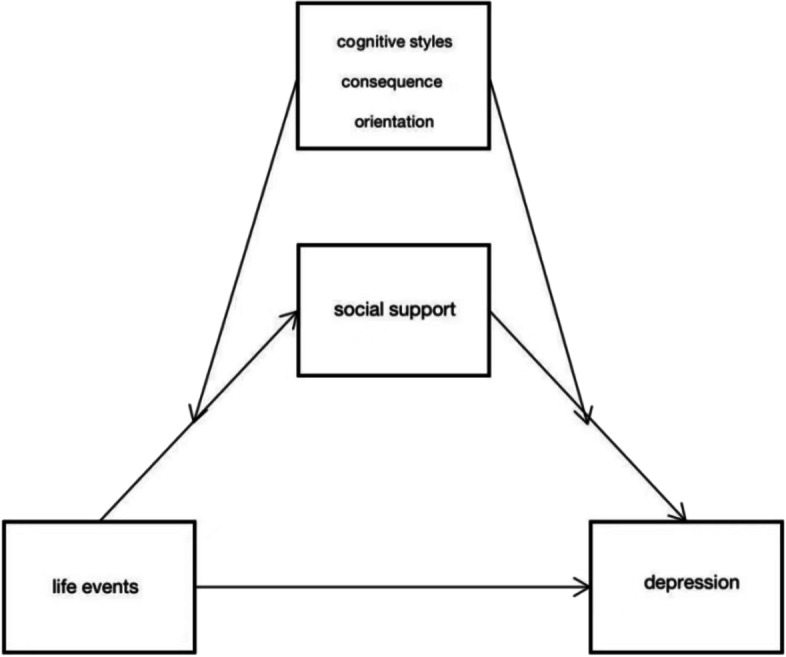
Conceptual framework

Based on the above theories and research, we have gained a preliminary understanding of life events, social support, depression, and cognitive styles. The purpose of this study is to verify whether the relationship between negative life events and depression experienced by adolescents in Chongqing is mediated by social support and moderated by cognitive styles. Based on the literature review, we established a theoretical hypothesis model (shown in Figs. 1, 2 and 3) and proposed the following 3 hypotheses:

- H1: Life events positively predict depressive symptoms in children and adolescents.
- H2: Life events can indirectly predict depressive symptoms in children and adolescents through the mediating role of social support.
- H3: The indirect effect of life events on depression via social support is moderated by cognitive styles and their two orientations: self-orientation and consequence orientation, and it’s a two-stage moderated mediation model. Specifically, the effect of life events on social support is weaker for children and adolescents with more passive cognitive styles than for those with more positive cognitive styles both in the self-orientation and the consequence orientation. At the same time, the effect of social support on depression is stronger for children and adolescents with more passive cognitive styles than for those with more positive cognitive styles both in the self-orientation and the consequence orientation.

## Method

### Participants and recruitment

With the assistance of three governmental agencies, the Chongqing Office of Poverty Alleviation and Development, Chongqing Foundation for Poverty Alleviation, and Chongqing Municipal Education Commission, we conducted a survey in the Shapingba District, Chongqing. The study was conducted according to the guidelines of the Declaration of Helsinki and was reviewed and approved by the Medical Ethics Committee of the Army Medical University (No. CWS20J007). We contacted the parents or legal guardians of the participants and asked for their consent to enroll their children in the study. We recruited 20 professionally trained graduates and teachers as data collection investigators, all of whom were trained in how to conduct the questionnaire and answer questions from the participants. After reading the informed consent, the subjects could choose whether to conduct the study or not according to their own will. Meanwhile, the subjects were guaranteed that all contents of the study would be conducted anonymously and no personal information would be disclosed in the study.

A total of 3,540 children and adolescents from 24 primary and middle schools in the Shapingba District of Chongqing were surveyed. The researcher selected 24 primary schools and middle schools in the Shapingba District by stratified randomized sampling, and randomly selected several classes in each grade from grade 3 to grade 12 for investigation. We collected 3540 questionnaires at first and a total of 2804 questionnaires were obtained by excluding those with missing, incomplete answers, contradictory answers, multiple choices and inaccurate data filling. The questionnaire recovery rate was 79.5%. All subjects completed the sociodemographic Information Form, Childhood Depression Inventory (CDI), Adolescent Self-Rating Life Events Checklist (ASLEC), Children’s Cognitive Style Questionnaire (CCSQ) and Child and Adolescent Social Support Scale (CASSS).

### Measures

#### Sociodemographic Information Form (SIF)

We collected sociodemographic information about the participants including gender, age, grade, only-child, boarding school and so on.

#### Childhood Depression Inventory (CDI)

The Chinese version of the Child Depression Scale [52] consisted of 27 self-reported questions about the cognitive, emotional and behavioral symptoms of children's depression was adopted. It includes five dimensions: anhedonia, negative emotion, low self-esteem, inefficiency and interpersonal problems. Each item on the scale had three options describing different levels of depressive symptoms, which were counted as 0, 1 and 2 respectively. Among them, 13 items were scored in reverse, with the total score ranging from 0 to 54, and the higher total score indicating a higher degree of depression. Previous studies have shown that the Chinese version of the CDI scale has good reliability and validity in Chinese children and adolescents [53]. In this study, the internal consistency of scales was good (Cronbach's α = 0.843).

#### Adolescent Self-Rating Life Events Checklist (ASLEC)

Life events of children and adolescents were measured using the Chinese version of the Adolescent Self-Rating Life Events Checklist [54]. There are 27 questions that can be divided into 6 dimensions. The scale summarizes the common life event stressors of children and adolescents and they are interpersonal relationship, study pressure, punishment, loss of relatives, friends and property, health and adaptation, and others. The participants were asked to self-report whether any of the life events listed in the options had occurred in the last 3 months. Apart from collecting data on occurrence, further evaluation of its impact was investigated, with a score ranging from 1 to 5, where “1” meant no impact and “5” meant very serious impact. A high score indicated a high influence of stressful life events. Previous studies have shown that this scale has good reliability and validity in the Chinese population [54]. The internal consistency of scales in this study was good (Cronbach's α = 0.914).

#### Child and Adolescent Social Support Scale (CASSS)

The Chinese version of the Child and Adolescent Social Support Scale was used to measure social support [55]. The scale has 60 questions and consists of 5 sub-scales, involving 5 dimensions that constitute the sources of social support for children and adolescents, namely, parents, classmates, teachers, friends and school people. Each subscale can be used independently, and each has 12 questions. Questions 1 to 3 of each subscale measured emotional support, questions 4 to 6 measured informational support, questions 7 to 9 measured evaluative support, and questions 10 to 12 measured instrumental support. Each question is scored according to the frequency of action, which is scored on a 6-point scale, with 1 meaning never and 6 meaning always. Previous studies have shown that this scale has good reliability and validity in China [56]. In this study, the internal consistency of the scale was good (Cronbach's α = 0.964).

#### Children’s Cognitive Style Questionnaire (CCSQ)

We used the Chinese version of the children's cognitive style questionnaire to measure the negative cognitive style of left-behind children. It was comprised of two subscales: consequence orientation scale and self-orientation scale. The former assesses the tendency to catastrophize the consequences of negative events, while the latter assesses the tendency to view oneself as flawed or deficient following negative events [48]. Each part contains 12 items and each part represents one aspect of a negative cognitive style [49]. In the consequence orientation scale, each item was rated from 1 to 4, with “1” meaning “it will not let me incur any other bad things” and “4” meaning “it will let me incur something terrible”. In the self-orientation scales, the score ranged from 1 to 3, with “1” meaning “it doesn't make me feel bad about myself” and “3” means "it makes me feel bad about myself”. Previous studies have shown that this scale has good reliability [57]. The internal consistency of the two subscales in this study was good, and Cronbach's α was 0.842 and 0.778, respectively.

### Statistical analyses

SPSS 26.0 was used for the statistical analysis of the data. We first carried out descriptive statistics on demographic variables. Then descriptive statistics and Pearson correlation analysis were performed for the four variables. We standardized the variable data. The SPSS Macro PROCESS 58 model was used for moderated mediation analysis. We first studied the influence mechanism of life events on children and adolescents' depression and explored the mediating model of social support. Secondly, the moderating effects of cognitive styles on the life events to social support and social support to depression pathways were tested.

### Results

#### Common method biases tests

To examine common method biases and systematic errors due to self-rating questionnaires, the study conducted Harman's single-factor test [58] and exploratory factor analysis for all items containing four variables. The results showed that the first factor accounted for 16.41% of the total variation, lower than the threshold of 40% proposed by Podsakoff et al. [59]. Although this result does not eliminate the possibility of common method variance, it suggests that common method bias is unlikely to confuse the interpretation of data analysis results.

### Descriptive statistics

The demographic data of the cases is presented in Table 1. Among all the participants, 48.6% are boys and 51.4% are girls. The participants’ ages ranged from 7 to 19 years (mean = 13.40, SD = 2.94). 68.2% of children and adolescents were from urban areas, while the rest 31.8% were from rural villages. Among all children and adolescents, only-child accounted for 62.8%, while non-single child 37.2%. 39.3% of respondents were from boarding schools, while the rest were from non-boarding schools.

**Table 1:** Descriptive statistics

Variable	N	(%)	Variable	N	(%)
**Age**			**Gender**		
7	2	0.1	Boys	1362	48.6
8	161	5.7	Girls	1442	51.4
9	133	4.7	**Only-child**		
10	250	8.9	Yes	1760	62.8
11	408	14.6	No	1044	37.2
12	155	5.5	**Boarding school**		
13	193	6.9	Yes	1102	39.3
14	380	13.6	No	1702	60.7
15	253	9			
16	344	12.3			
17	366	13.1			
18	155	5.5			
19	4	0.1			

### Descriptive analysis and correlations between overall variables

The basic descriptive data for life events, depression, social support and cognitive styles are shown in Table 2. The mean total scores for life events were 32.83 ± 21.78 (range = 0–135), and the mean total scores for CDI were 12.57 ± 7.03 (range = 0–52), the mean total scores for social support were 221.03 ± 47.33 (range = 60–360), the mean total scores for cognitive styles were 47.11 ± 9.36 (range = 24–84), and the mean total scores of the self-orientation cognitive styles were 21.02 ± 4.47 (range = 12–36), the mean total scores of the consequence orientation cognitive styles were 26.09 ± 6.03 (range = 12–48).

**Table 2:**
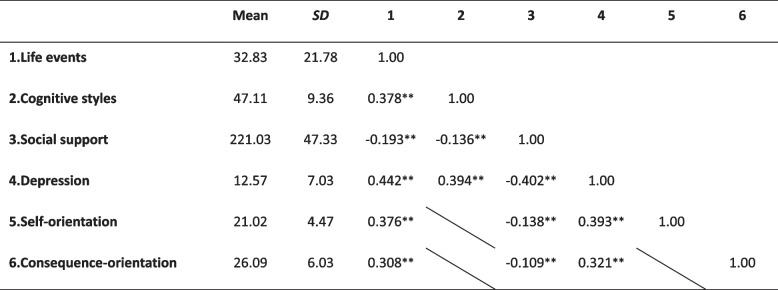
Descriptive analysis and correlations

*N *= 2804

**p< 0.01; All tests were two-tailed. This table shows the general means, standard deviations, and correlations of the six major variables. **indicates a significant correlation between the variables, which obtains between all the variables

Pearson correlation analysis was used to test the bivariate correlations of all the variables. All variables in the model have been standardized. Table 2 shows that all the variables were significantly correlated with each other. Life events were significantly positively correlated with cognitive styles and their two orientations and negatively correlated with social support (*r* = 0.37, *p* < 0.01; *r* = 0.38, *p* < 0.01; *r* = 0.31, *p* < 0.01; *r* = -0.19, *p* < 0.01). Cognitive styles were significantly negatively correlated with social support (*r* = -0.14, *p* < 0.01). Depression was significantly and positively correlated with life events, cognitive styles and its two orientations were negatively correlated with social support (*r* = 0.31, *p* < 0.01; *r* = 0.39, *p* < 0.01; *r* = 0.39, *p* < 0.01; *r* = 0.32, *p* < 0.01; *r* = -0.40, *p* < 0.01).

### Mediation analysis

Life events, cognitive styles and social support were significantly correlated, which met the statistical requirements for further mediating effect analysis of life events and depression [60]. After controlling for gender, age, only-child and boarding school, Model 4 in SPSS 26.0 compiled by Hayes was used to analyze the mediating effect of social support in the relationship between life events and depression.

Table 3 shows the mediating effect value of social support in the relationship between life events and depression. Life events were directly and positively predictive of depression (b = 0.3679, *p* < 0.001) and were negatively associated with social support (b = -0.1895, *p* < 0.001), which in turn was negatively related to depression (b = -0.3318, *p* < 0.001). The bootstrapped 95% CI confirmed the significant indirect effects of social support in the relationship between life events and depression (b = 0.06280, 95% CI [0.0483, 0.0782]). The total effect value of life events on depression was 0.431, the direct effect value of life events on depression was 0.368 and the total standardized mediating effect value was 0.063. The ratio of the total standardized mediating effect to the total effect was 14.62%. The indirect effects reached a significant level because the 95% confidence interval of the above indirect effects did not contain the zero value. These results indicated that social support partially mediated the relationship between life events and depression.

**Table 3:** Mediation Effect

Predictor	Model 1(Social support)					Model 2(Depression)		
	**B**	**SE**	***t***	***p***	**B**	**SE**	***t***	***p***
**Age**	-0.0533	0.0073	-2.5910	0.0096**	0.0401	0.0062	2.1914	0.0285*
**Gender**	-0.0896	0.0370	-4.8464	0.0000***	-0.0477	0.0318	-3.0042	0.0027**
**Only-child**	-0.0213	0.0385	-1.1448	0.2524	0.0084	0.0329	0.5249	0.5997
**Boarding school**	-0.0444	0.0442	-2.0557	0.0399*	-0.007	0.0378	-0.3811	0.7031
**Life events**	-0.1895	0.0192	-9.8834	0.0000	0.3679	0.0167	22.0607	0.000***
**Social support**					-0.3318	0.0162	-20.5199	0.000***
**R**^**2**^	0.0477				0.3034			
**F**	28.0334***				203.054***			

*p < 0.05, **p < 0.01, ***p < 0.001

### Moderated mediation analysis

After the mediation test, we further tested the moderated mediation by running a two-stage moderated mediation model in the PROCESS 3.4.1 Model 58. We found evidences for both first-stage and second-stage moderated mediation.

**Fig. 4:**
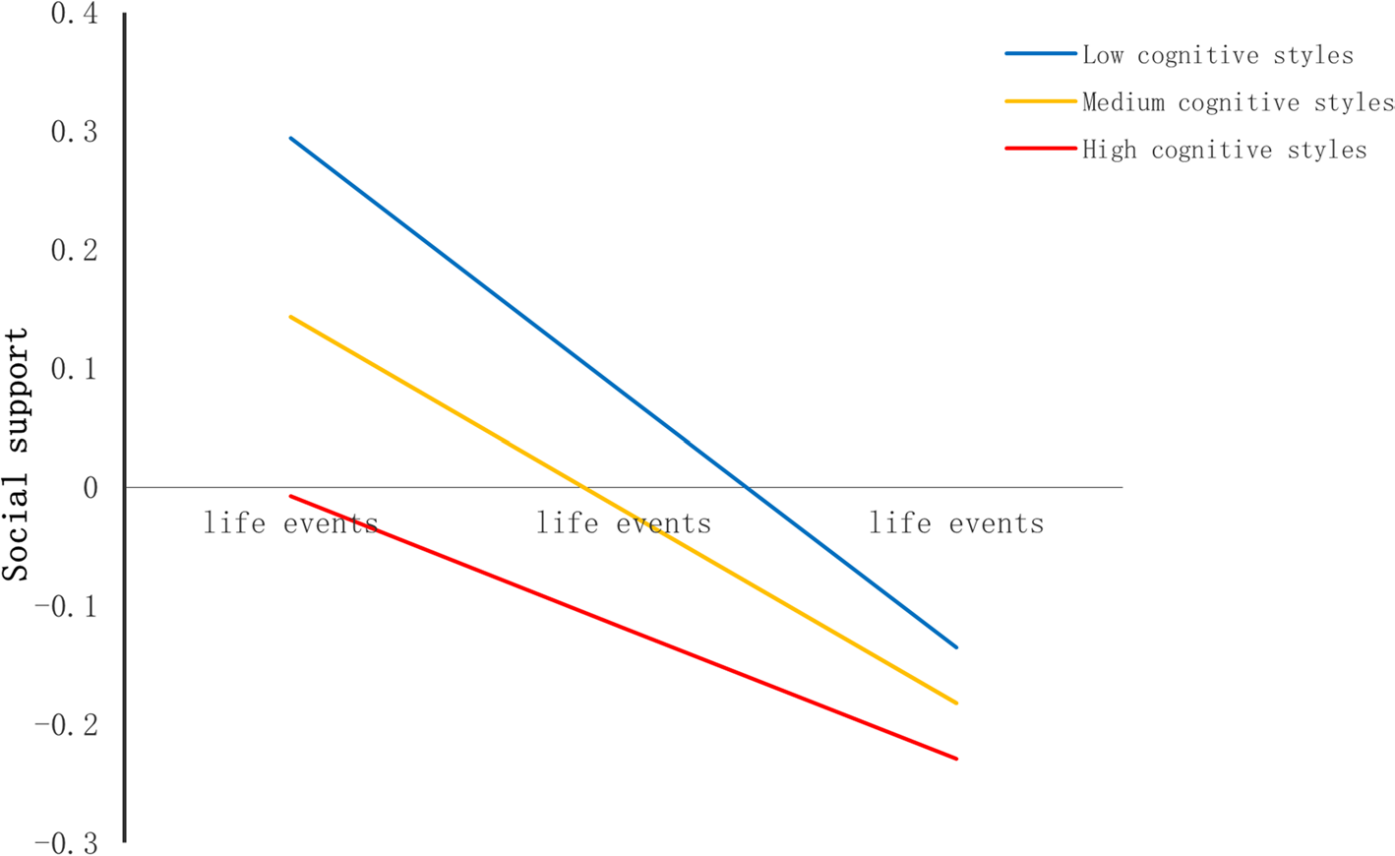
Cognitive styles as a moderator in the association between life events and social support

First, cognitive styles moderated the relationship between life events and social support (see Table 4). The shape of the moderation was described in Fig. 4. The conditional effect of life events on social support was strongest at lower levels of cognitive styles (− 1SD); (B = -0.21, SE(B) = 0.03, *p* < 0.001), moderate at mean level (B = -0.16, SE(B) = 0.02, *p* < 0.001), and weakest at higher (+ 1SD) levels (B = -0.11, SE(B) = 0.02, *p* < 0.001).

**Table 4:** Moderated Mediation Effect

Variables	B	SE	*t*	R^2^
**Outcome variable (Social support)**				
0.0588				
**Constant**	0.8362	0.1718	4.8680***	
**Age**	-0.0275	0.0075	-3.6538***	
**Gender**	-0.1993	0.0371	-5.3749***	
**Only-child**	-0.0286	0.0384	-0.7455	
**Boarding school**	-0.0950	0.0440	-2.1581	
**Life events**	-0.1626	0.0219	-7.4203***	
**Cognitive styles**	-0.0106	0.0022	-4.6994***	
**Life events × cognitive styles**	0.0055	0.0019	2.9558**	
**Outcome variable (Depression)**				
0.3684				
**Constant**	-0.4736	0.1411	-3.3568***	
**Age**	0.0422	0.0062	6.8506***	
**Gender**	-0.0305	0.0305	-1.0002	
**Only-child**	-0.0155	0.0314	-0.4933	
**Boarding school**	-0.0228	0.0360	-0.6320	
**Life events**	0.2478	0.0174	14.2192***	
**Social support**	-0.3088	0.0155	-19.9773***	
**Cognitive styles**	0.0308	0.0018	16.6358***	
**Social support × cognitive styles**	-0.0072	0.0015	-4.8833***	

*p < 0.05, **p < 0.01, ***p < 0.001

**Fig. 5:**
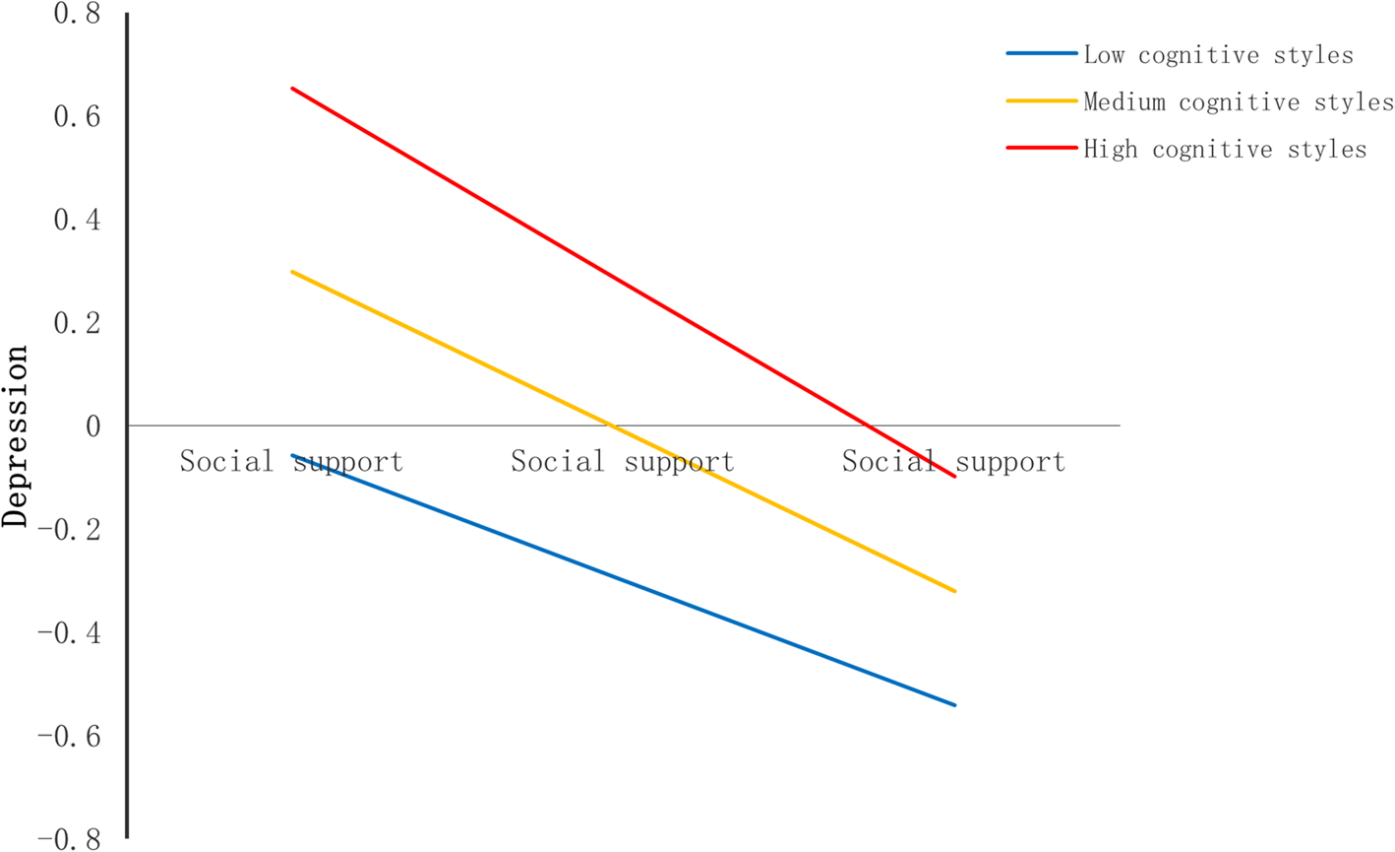
Cognitive styles as a moderator in the association between social support and depression

Second, we found that cognitive styles also moderated the relationship between social support and depression (see Table 4). The shape of the moderation was described in Fig. 5. The conditional effect of social support on depression was strongest at higher levels of cognitive styles (+ 1SD); (B = -0.38, SE(B) = 0.02, *p* < 0.001), moderate at mean level (B = -0.31, SE(B) = 0.02, *p* < 0.001), and weakest at lower (+ 1SD) levels (B = -0.24, SE(B) = 0.02, *p* < 0.001).

**Fig. 6:**
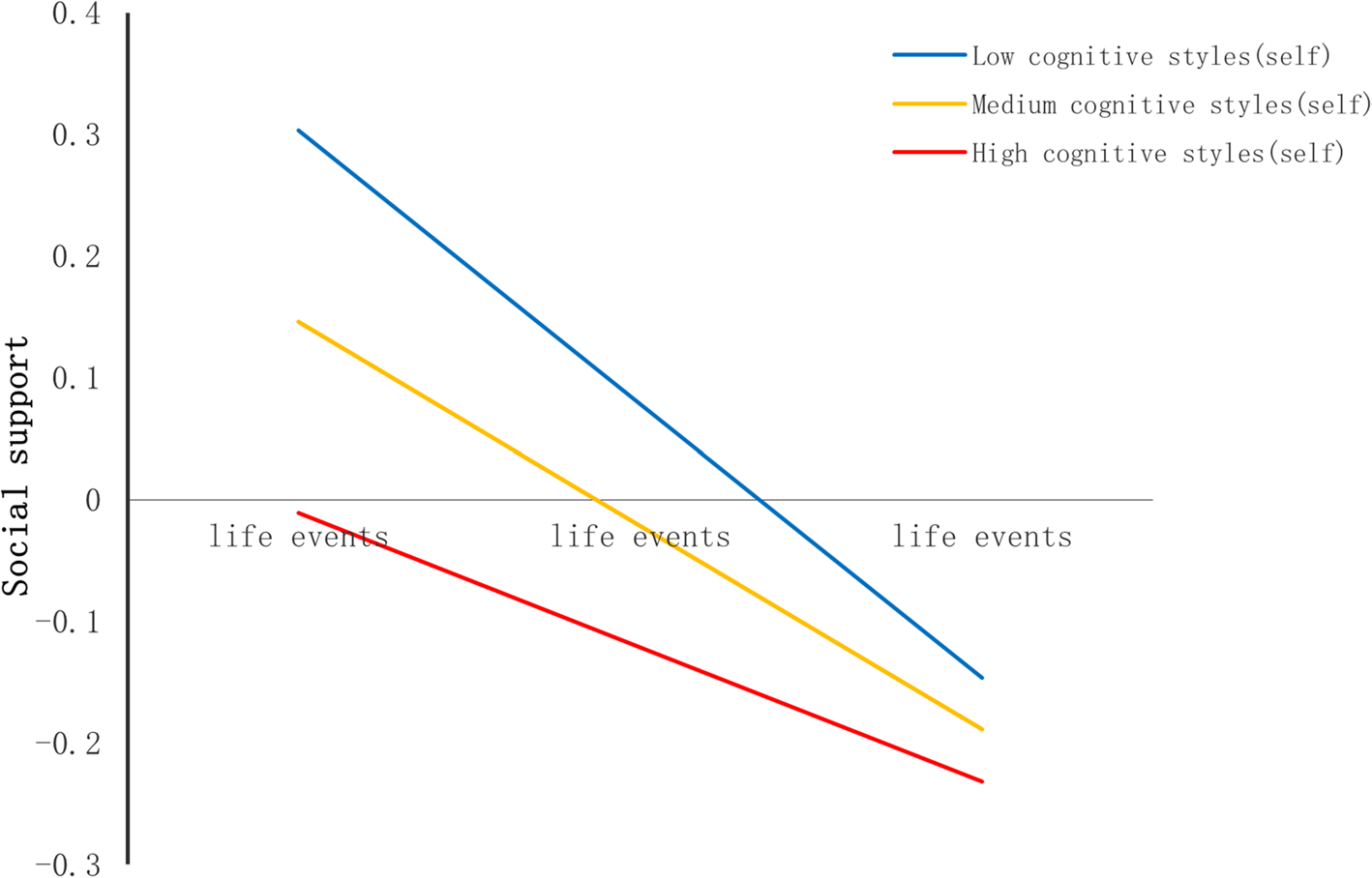
Cognitive styles (self) as a moderator in the association between life events and social support

**Fig. 7:**
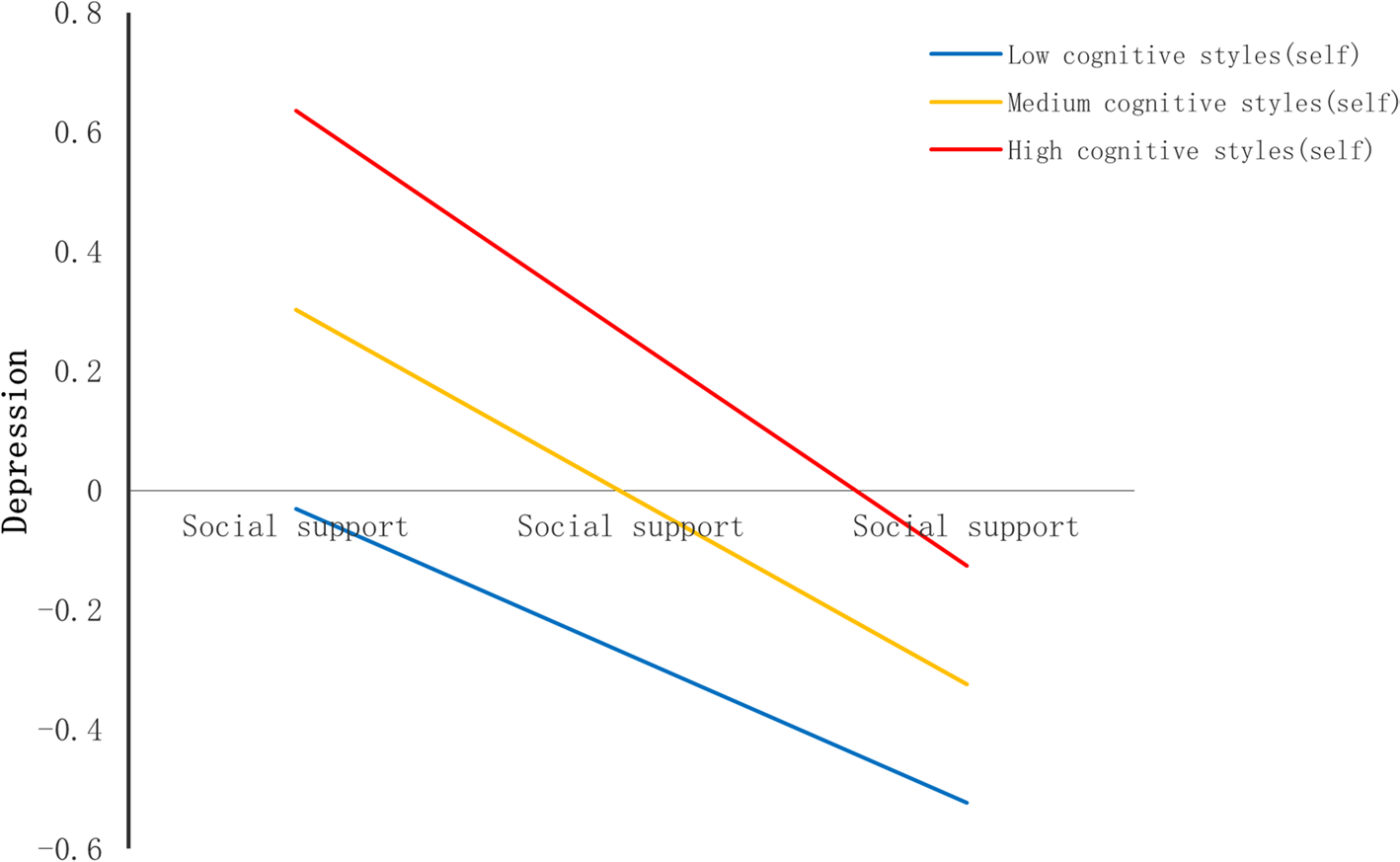
Cognitive styles (self) as a moderator in the association between social support and depression

**Fig. 8:**
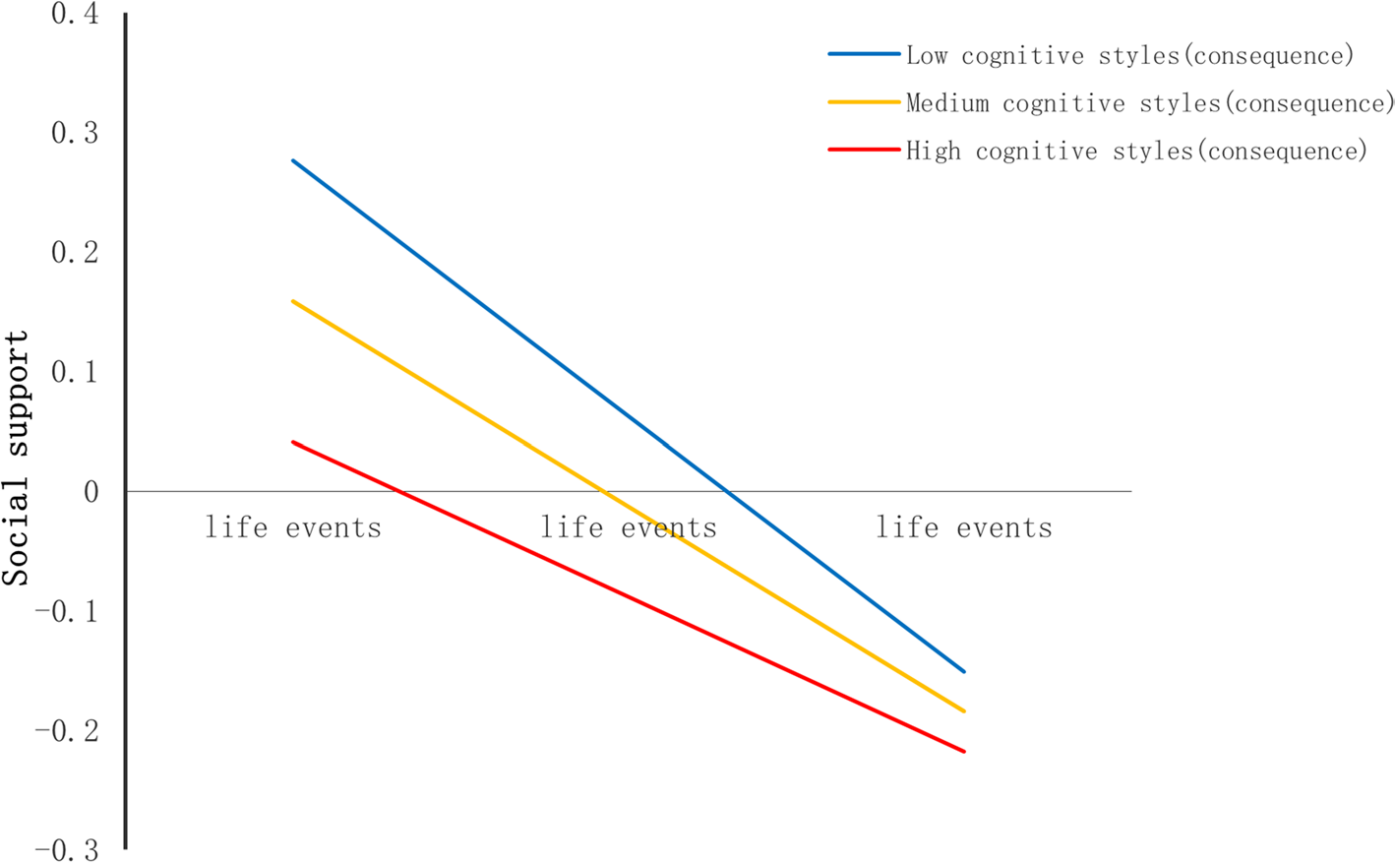
Cognitive styles (consequence) as a moderator in the association between life events and social support

**Fig. 9:**
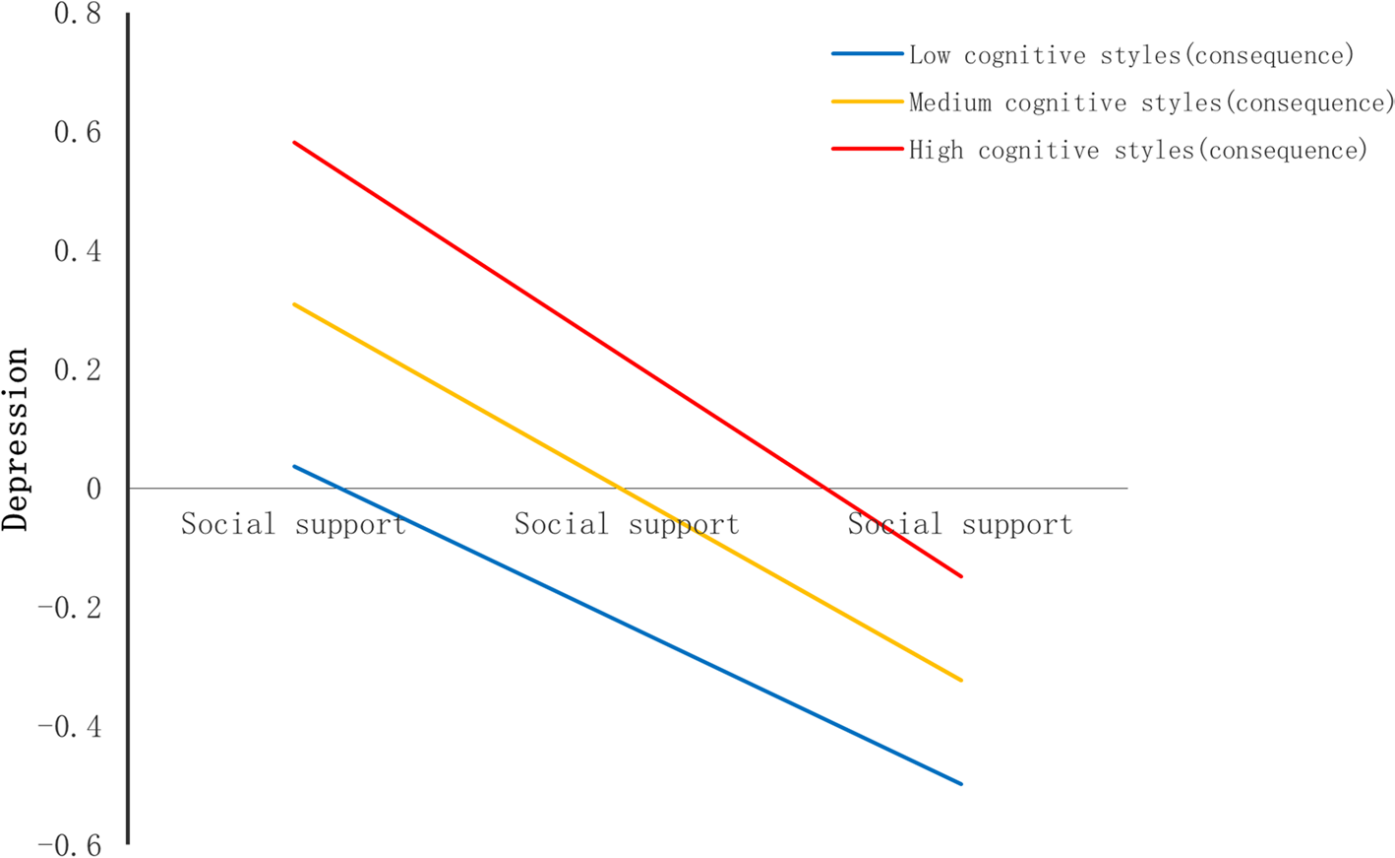
Cognitive styles (consequence) as a moderator in the association between social support and depression

After confirming the moderating effect of cognitive styles, we further tested the moderating effect of two cognitive style orientations (self-orientation and consequence orientation). In the self-orientation, we found moderated mediations for both first stage (see Table 5, Fig. 6.) and second stage (see Table 5, Fig. 7). In the consequence orientation, we also found moderated mediations for both first stage (see Table 6, Fig. 8) and second stage (see Table 6, Fig. 9).

**Table 5:** Moderated Mediation Effect

Variables	B	SE	*t*	R^2^
**Outcome variable (Social support)**				
0.0596				
**Constant**	0.8258	0.1710	4.8297***	
**Age**	-0.0257	0.0074	-3.4760***	
**Gender**	-0.1978	0.0370	-5.3466***	
**Only-child**	-0.0298	0.0384	-0.7769	
**Boarding school**	-0.1045	0.0440	-2.3738	
**Life events**	-0.1676	0.0215	-7.7804***	
**Cognitive styles (self)**	-0.0224	0.0046	-4.8832***	
**Life events × cognitive styles (self)**	0.0128	0.0038	3.3675***	
**Outcome variable (Depression)**				
0.3633				
**Constant**	-0.4014	0.1411	-2.8445**	
**Age**	0.0349	0.0061	5.7234***	
**Gender**	-0.0405	0.0306	-1.3244	
**Only-child**	-0.0148	0.0315	-0.4700	
**Boarding school**	0.0020	0.0362	0.0540	
**Life events**	0.2630	0.0173	15.2336***	
**Social support**	-0.3130	0.0155	-20.1439***	
**Cognitive styles (self)**	0.0595	0.0038	15.6909***	
**Social support × cognitive styles(self)**	-0.0151	0.0032	-4.7937***	

*p < 0.05, **p < 0.01, ***p < 0.001

**Table 6:** Moderated Mediation Effect

Variables	B	SE	*t*	R^2^
**Outcome variable (Social support)**				
0.0546				
**Constant**	0.7984	0.1714	4.6573***	
**Age**	-0.0251	0.0075	-3.3557***	
**Gender**	-0.1925	0.0371	-5.1923***	
**Only-child**	-0.0335	0.0384	-0.8713	
**Boarding school**	-0.0887	0.0441	-2.0100	
**Life events**	-0.1714	0.0210	-8.1673***	
**Cognitive styles (consequence)**	-0.0125	0.0034	-3.6968***	
**Life events × cognitive styles (consequence)**	0.0070	0.0029	2.3861*	
**Outcome variable (Depression)**				
0.3445				
**Constant**	-0.3384	0.1431	-2.3642*	
**Age**	0.0348	0.0062	5.5753***	
**Gender**	-0.0515	0.0310	-1.6601	
**Only-child**	-0.0023	0.0320	-0.0729	
**Boarding school**	-0.0348	0.0367	-0.9462	
**Life events**	0.2885	0.0173	16.7044***	
**Social support**	-0.3160	0.0157	-20.0966***	
**Cognitive styles (consequence)**	0.0371	0.0028	13.0669***	
**Social support × cognitive styles(consequence)**	-0.0081	0.0023	-3.5045***	

*p < 0.05, **p < 0.01, ***p < 0.001

In self-orientation, cognitive styles moderated the relation between life events and social support (see Table 5). The shape of the moderation was described in Fig. 6. The conditional effect of life events on social support was strongest at lower levels of cognitive styles (self-orientation) (− 1SD); (B = -0.225, SE(B) = 0.030, *p* < 0.001), moderate at mean level (B = -0.167, SE(B) = 0.022, *p* < 0.001), and weakest at higher (+ 1SD) levels (B = -0.110, SE(B) = 0.024, *p* < 0.001). What’s more, the cognitive styles (self-orientation) also moderated the relation between social support and depression (see Table 5). The shape of the moderation was described in Fig. 7. The conditional effect of social support on depression was strongest at higher levels of cognitive styles (self-orientation) (+ 1SD); (B = -0.381, SE(B) = 0.022, *p* < 0.001), moderate at mean level (B = -0.313, SE(B) = 0.016, *p* < 0.001), and weakest at lower (+ 1SD) levels (B = -0.246, SE(B) = 0.021, *p* < 0.001).

In the consequence orientation, cognitive styles moderated the relation between life events and social support (see Table 6). The shape of the moderation was described in Fig. 8. The conditional effect of life events on social support was strongest at lower levels of cognitive styles (consequence orientation) (− 1SD); (B = -0.213, SE(B) = 0.030, *p* < 0.001), moderate at mean level (B = -0.171, SE(B) = 0.021, *p* < 0.001), and weakest at higher (+ 1SD) levels (B = -0.129, SE(B) = 0.024, *p* < 0.001). And the cognitive styles (consequence orientation) also moderated the relationship between social support and depression (see Table 6). The shape of the moderation was described in Fig. 9. The conditional effect of social support on depression was strongest at higher levels of cognitive styles (consequence orientation) (+ 1SD); (B = -0.365, SE(B) = 0.021, *p* < 0.001), moderate at mean level (B = -0.316, SE(B) = 0.016, *p* < 0.001), and weakest at lower (+ 1SD) levels (B = -0.267, SE(B) = 0.021, *p* < 0.001).

## Discussions and conclusions

This study investigated the relationship between life events and depression and the mediating role of social support and the moderating effect of cognitive styles among Chinese adolescents and children. The results show that social support has a partial mediating effect on the relationship between life events and depression in children and adolescents, and cognitive styles can not only moderate the relationship between life events and social support, but also the relationship between social support and depressive symptoms. This will help us further understand the mechanism of the relationship between life events and depression, and guide the government and relevant authorities to improve the mental health status of children and adolescents.

The study first examined the relationship between life events and depression in children and adolescents. When children and adolescents experienced more negative stressful life events, they showed higher levels of depression. This is also consistent with previous findings [13, 24]. In the current educational environment in China, a heavy academic burden falls on children and teenagers. In addition, they will encounter many key stages in their growth process, and stressful life events from various aspects of life will have a great impact on their mental health [61].

After identifying the relationship between life events and depression, we further validated the mediating role of social support in the relationship between life events and depression in children and adolescents. This is consistent with previous findings about the mediating role of social support in adolescents’ life events and depression [34]. Social support has long been used as a protective factor for depression, and in recent years many studies have examined the role of social support in depression, but these studies only considered it as a moderating factor that significantly reduces depression in children and adolescents [62, 63]. However, the mediating role of social support in depression can not be ignored. Studies have shown that life events can change adolescents' attachment style [64], and adolescents' attachment style is directly related to social support [65]. Compared with normal attachment individuals, individuals with avoidant attachment tend to contact others less, resulting in less perceived social support. Meanwhile, according to stress buffering theory, social support is an important tool for reducing stress, and perceived social support can help us reduce the impact of negative stimuli. At the same time, social support, as an external resource, can promote post-stress growth, thereby helping individuals improve their overall mental health [66] and enhance psychological regulation [67]. This is also consistent with previous studies that social support plays a mediating role between stress and mental health [41], and social support plays a mediating role in perceived stress and post-stress growth [68]. These studies also further support the possibility that social support plays a mediating role in life events and depression. Our results also demonstrate that when children and adolescents experience more life events, they experience less social support, which in turn increases the onset of depression in the absence of this protective factor. The results show the importance of social support in the development of children and adolescents. Social support is mainly derived from parents, teachers, classmates, friends and other important caregivers, among which family education and school education are the most important. The results show that the active care and support from parents and teachers are extremely important in the mental health of children and adolescents. Many children choose to face the pressure of life alone, which will worsen their mental health in the long run. Social support, as a protective factor, can improve this relationship and take better care of children's growth.

In addition, this study also verified the two-stage moderating effects of cognitive styles and their two sub-dimensions of self-orientation and consequence orientation. Over the past few decades, there has been ample research on the relationship between negative cognitive styles and depression. Negative cognition has been found to be a risk factor for depression. Under the model of cognitive depression, the negative cognitive pattern produced by individuals due to environmental pressure will have a huge impact on individuals' lives [44]. The results show that cognitive styles can moderate the relationship between life events and social support, that is, a positive cognitive style can alleviate the negative impact of life events on perceived social support, while a negative cognitive style can further aggravate the impact. This further verifies the previous conclusion that life events interact with cognitive function [49], and that the accumulation of life events can impair individual cognition and lead to more depressive symptoms. At the same time, cognitive styles can also moderate perceived social support and depression, that is, a positive cognitive style can further strengthen the protective effect of social support on depression, while a negative cognitive style can inhibit and reduce the protective effect. This conclusion is consistent with the previous research [51]. The results show that cognitive styles play an important role in depression. Also, our study pioneered the exploration of two dimensions of cognitive styles: self-orientation and consequence orientation. From the results, we can see that cognitive styles have a moderating effect on both the self-orientation and consequence orientation. By definition, we know that individuals with the self-orientation cognitive style tend to think of themselves as flawed and individuals with the consequence orientation cognitive style would view what happens as negative and catastrophic. Although we did not explore it in this study, based on previous research [48], we can know that the self-orientation cognitive style is more closely related to social support. Individuals with a self-orientation cognitive style see themselves as flawed and therefore are more likely to blame themselves and get caught up in negative emotions when faced with negative events in their lives. The consequence orientation cognitive style, on the other hand, is more related to life events. Individuals with a consequence orientation cognitive style, when confronted with life events, tend to perceive them as bad and disastrous, thus impairing their mental health. According to this result, teachers and parents can develop children's positive cognitive style from these two dimensions in future school and family education. Individuals with positive cognitive styles can reduce the negative effect of life events on depression in many ways, while individuals with negative cognitive styles will aggravate the negative effect on the original basis and bring more depression. This also tells us that it is extremely important to develop positive cognitive styles in children and adolescents. Cognitive patterns are formed early in life and persist throughout life. Children and teenagers are in this critical stage, family education and school education must pay attention to the cultivation of children's cognition, establish a positive cognitive style will postively influence the children's mental health in the future.

Our results confirm that life events and negative cognitive styles are risk factors for depressive symptoms in children and adolescents, and that social support is a protective factor for depression, which is consistent with previous studies [13]. The mental health problem of children and adolescents is a hot issue in today's society. In recent years, the relevant “double reduction” policy to reduce students' burden of homework and off-campus training at the compulsory education level issued by the central government is aimed at better relieving the pressure of children. Admittedly, every child will encounter more or less stressful events as they grow up, and it is difficult for us to change these events, but we can intervene the pathways between life events and depression and try to change these variables to reduce depressive symptoms. Our study found that social support plays a mediating role, and cognitive style plays a moderating role in both mediation pathways. This also emphasizes the importance of developing strong social support for children and adolescents. When children grow up, some of them may not actively seek social support. At this time, parents, schools and relevant institutions need to pay more attention to the growth of children, pay attention to their life, care about them and accompany them, which can improve their psychological condition to some extent. Besides, schools and parents should also pay attention to cultivating positive cognitive styles in children. We can see that under the same pressure, the effects of different cognitive styles are significantly different. Stress and frustration are inevitable in everyone's life. Having a positive cognitive style and actively coping with pressure will make individuals psychologically healthier in the face of adversity.

There are some limitations in this study. First of all, this study is a cross-sectional study, which only shows the correlation of variables rather than causality. Future studies can use longitudinal research methods to further explore the relationship between variables. Secondly, all the questionnaires used in this study are self-reported. The questionnaire involves many real-life situations. Although there are instructions, the results of the questionnaire may be affected by the errors caused by social prejudice and the self-defense mechanism of the participants. Subsequent studies can start from peer evaluation, teacher evaluation, parent evaluation and other perspectives. Thirdly, the sample selected in this study were children and adolescents from one district of Chongqing, so the results have certain limitations, and the follow-up research can expand the scope of the investigation. Finally, this study only discussed the mediating relationship between these variables, but did not conduct a more detailed analysis of the influence of some potential moderating variables on depression, such as gender and age. Subsequent studies could focus on the moderating effect of demographic variables.

Despite these limitations, this study is the first to examine the relationship between life events, social support, depression, and cognitive styles in children and adolescents. Unlike previous studies that only considered social support as a moderating variable, we tested the possibility that social support is a mediating variable. At the same time, we also discovered the moderation effects of the cognitive styles and the two-stage moderation of its two dimensions. The results also support our hypothesis that life events positively predict depressive symptoms in children and adolescents. At the same time, life events can indirectly predict the depressive symptoms of children and adolescents through the mediating role of social support, and cognitive styles can moderate the relationship between life events and social support and depression.

## Data Availability

The datasets generated during the current study are not publicly available due to the reason that we didn't list the release of the data when we signed the consent form, but are available from the corresponding author on reasonable request.
